# Prediction of left ventricular ejection fraction changes in heart failure patients using machine learning and electronic health records: a multi-site study

**DOI:** 10.1038/s41598-023-27493-8

**Published:** 2023-01-06

**Authors:** Prakash Adekkanattu, Luke V. Rasmussen, Jennifer A. Pacheco, Joseph Kabariti, Daniel J. Stone, Yue Yu, Guoqian Jiang, Yuan Luo, Pascal S. Brandt, Zhenxing Xu, Veer Vekaria, Jie Xu, Fei Wang, Natalie C. Benda, Yifan Peng, Parag Goyal, Faraz S. Ahmad, Jyotishman Pathak

**Affiliations:** 1 Weill Cornell Medicine, New York City, NY USA; 2 Northwestern University, Chicago, IL USA; 3 The Mayo Clinic, Rochester, MN USA; 4 University of Washington, Seattle, WA USA; 5 University of Florida, Gainesville, FL USA

**Keywords:** Machine learning, Health care, Heart failure

## Abstract

Left ventricular ejection fraction (EF) is a key measure in the diagnosis and treatment of heart failure (HF) and many patients experience changes in EF overtime. Large-scale analysis of longitudinal changes in EF using electronic health records (EHRs) is limited. In a multi-site retrospective study using EHR data from three academic medical centers, we investigated longitudinal changes in EF measurements in patients diagnosed with HF. We observed significant variations in baseline characteristics and longitudinal EF change behavior of the HF cohorts from a previous study that is based on HF registry data. Data gathered from this longitudinal study were used to develop multiple machine learning models to predict changes in ejection fraction measurements in HF patients. Across all three sites, we observed higher performance in predicting EF increase over a 1-year duration, with similarly higher performance predicting an EF increase of 30% from baseline compared to lower percentage increases. In predicting EF decrease we found moderate to high performance with low confidence for various models. Among various machine learning models, XGBoost was the best performing model for predicting EF changes. Across the three sites, the XGBoost model had an F1-score of 87.2, 89.9, and 88.6 and AUC of 0.83, 0.87, and 0.90 in predicting a 30% increase in EF, and had an F1-score of 95.0, 90.6, 90.1 and AUC of 0.54, 0.56, 0.68 in predicting a 30% decrease in EF. Among features that contribute to predicting EF changes, baseline ejection fraction measurement, age, gender, and heart diseases were found to be statistically significant.

## Introduction

Left ventricular ejection fraction (EF) is a critical measurement used in the diagnosis, prognosis, and treatment of patients with heart failure (HF). It compares the ratio of amount of blood pumped out to the total blood in the left ventricle of the heart. Historically, patients with HF have been categorized into two phenotypes based on their EF measurements: heart failure with reduced ejection fraction (HFrEF) with an EF value less than 50%, and heart failure with preserved ejection fraction (HFpEF) with an EF value equal to or greater than 50%. However, in recent years researchers have been exploring the characteristics and outcomes of a third borderline category called heart failure with mid-range ejection fraction (HFmrEF) with EF between 40 and 49% per the United States guidelines and between 41 and 49% per European guidelines^1,2^. Recent studies on HFmrEF patients showed some important distinctions from those patients with HFrEF and HFpEF^3^. All three phenotypes have been characterized by different pathologies^4–6^. Clinical practice guidelines recommend repeated EF measurements when there is a clinical change or a need to assess response to therapy^7^. US guidelines now recommend a fourth subphenotype of HFimpEF for those patients with a previous EF ≤ 40% and a follow-up measurement of EF > 40%^1^. Observation of longitudinal EF change has been reported to be an important factor in the prognosis and treatment options of HF^8,9^. An increase in EF correlates with improved long-term prognosis in patients with dilated cardiomyopathy^10^. Conversely, EF reduction is an important indicator of poor outcomes in those with drug-induced congestive HF^11^. Understanding the clinical implications of serial changes in EF may help guide the frequency of measurement, anticipate individual patient responses to evidence-based therapy, and augment existing risk model calculators.

Several studies have reported on the etiology, pathophysiology, and clinical characteristics of patients in HF subphenotypes based on their EF assessment at diagnosis^12^. A few studies have reported on EF changes that occur over time in patients diagnosed with HF^13,14^. By studying patients with two or more EF measurements in a HF registry, Savarese et al. recently analyzed the prevalence, prognostic implications, and predictors of all-cause mortality and/or HF hospitalizations in relation to EF changes^14^. Clinical trials have demonstrated an improvement in EF in some patients with HFrEF in response to the use of medications such as β blockers^15^. However, follow-up in these patients is often limited, and data is limited in studying changes in EF over time in patients with HFpEF^13^. While these studies support the hypothesis that serial EF measurements have predictive prognostic value, none of them have (1) been performed in a large patient cohort with an extensive number of clinical events, (2) featured a systematic approach to timing of EF measurements, (3) employed a consistent method of EF assessment, or (4) investigated changes in EF by race or sex^16^.

The widespread adoption of electronic health records (EHR) has raised the possibility of using such data in clinical research. Unlike data acquired in clinical trials, which is robustly collected but often limited in scope and specific to certain research objectives, EHR data represents a patient’s complete health trajectory, including demographics, vitals, diagnosis, labs, procedures, and medications and their response. This has resulted in a dramatic increase in clinical research using EHR data, especially for phenotyping and cohort identification^17^. There have been some efforts in recent years on HF phenotyping using EHR data^18–24^. These studies were mainly focused on identifying a cohort of patients satisfying one or more inclusion/exclusion criteria for HF. The above studies, however, did not explore HF subphenotypes (HFpEF, HFmrEF, and HFrEF) based on patients’ EF measurements. Similarly, those previous studies investigating EF changes in HF patients were conducted through HF registry data or site-specific clinical trials involving limited patient cohorts^13,14^. Despite the fact that EHR can support large-scale cohort analysis of HF patients, previously no study has been reported investigating changes in EF using clinical data from EHR. Machine learning (ML) techniques such as regression, clustering, decision trees, and support vector machines (SVM) can potentially identify patients at high risk for health conditions, such as heart disease, from EHR data^25^.

In this study, we used EHR data across three academic medical centers in the United States to investigate longitudinal changes of EF measurements in HF patients. We identified HF phenotypes following a previously validated algorithm^24^. We further identified subphenotypes of these HF patients based on EF measurements, and analyzed various characteristics including demographics, vitals, labs, procedures, and medications to determine if there are significant differences among these subphenotypes. We developed several machine learning models to predict EF changes in HF patients over a 1-year follow-up period following an initial EF measurement and identified major features that contribute to the performance of the prediction models. To the best of our knowledge, this is the first report on using machine learning to predict EF changes across a large cohort of HF patients from different health systems. We believe these models can form the foundation for any future clinical utility in accurately predicting changes in EF measurements for HF patients.

## Materials and methods

This multi-site study was conducted across three academic medical centers: Weill Cornell Medicine (New York, NY), Mayo Clinic (Rochester, MN), and Northwestern Medicine (Chicago, IL). We denote these sites as Site-A, Site-B, and Site-C in randomized order for anonymizing results from individual sites. The study was approved and the need for individual informed consent was waved by The Weill Cornell Medicine Institutional Review Board, The Northwestern University Institutional Review Board, and The Mayo Clinic Institutional Review Board. All methods were carried out in accordance with IRB and Health Insurance Portability and Accountability Act (HIPAA) guidelines. Weill Cornell Medicine (WCM) and its affiliate hospital NewYork-Presbyterian (NYP) now use the Epic EHR system in both inpatient and outpatient settings. Previously this was Epic Ambulatory instance for outpatient care and Allscripts in the inpatient setting from which the data for the current study was obtained. Information Technologies & Services manages several Microsoft SQL Server databases with regular data feeds from both outpatient and inpatient EHR systems. The Mayo Clinic uses a Unified Data Platform (UDP) to provide practical EHR data solutions and create a combined view of multiple heterogeneous data sources through effective data orchestration. Northwestern Medicine (NM) uses the Epic EHR system in both inpatient and outpatient settings and has an integrated enterprise data warehouse (EDW) to centralize access to clinical and ancillary data sources. We coded the target phenotype algorithm and covariates as SQL queries and retrieved data out of these EHR systems. Echocardiograms from site EHR systems were the main source for patients’ EF measurements which were available either natively as a structured data element or extracted from echo reports through natural language processing (NLP). At Cornell, EF measurements were extracted from various reports and procedures available in the EHR through NLP. These reports include Echocardiogram (LOINC codes: 42148-7, 59281-6, 85475-2, 59282-4), MRI abdomen (LOINC: 24557-1, 30668-8), MRI Abdomen-Pelvis [LOINC: 72246-2], and Myocardial perfusion study (LOINC: 9872-3). Details of the NLP extraction technique are described elsewhere^26,27^. The specific method for assessing LVEF varies in these procedures. Over 90% of EF measurements come from echocardiograms measured using varied methods (e.g. biplane, 3D, visual). Echo reports often contain both machine readout as well as the physician’s qualitative assessments on EF, and our NLP system captures both. Also at Northwestern Medicine, EF measurement was done using varied methods selected by the reader as the most accurate (e.g., biplane, visual, 3D), and was recorded either in clinical notes or discretely within Syngo. EF measurements not in a structured format were extracted using NLP, and all EF measurements were normalized to a single database table. At Mayo Clinic, Echocardiogram was the source of EF measurement. The UDP captures EF measurements as structured and recorded in several tables. In collecting EF measurements at the three sites, we made no distinction between EF measurements recorded during an outpatient visit and inpatient visit, and treated them as same.

**Figure 1:**
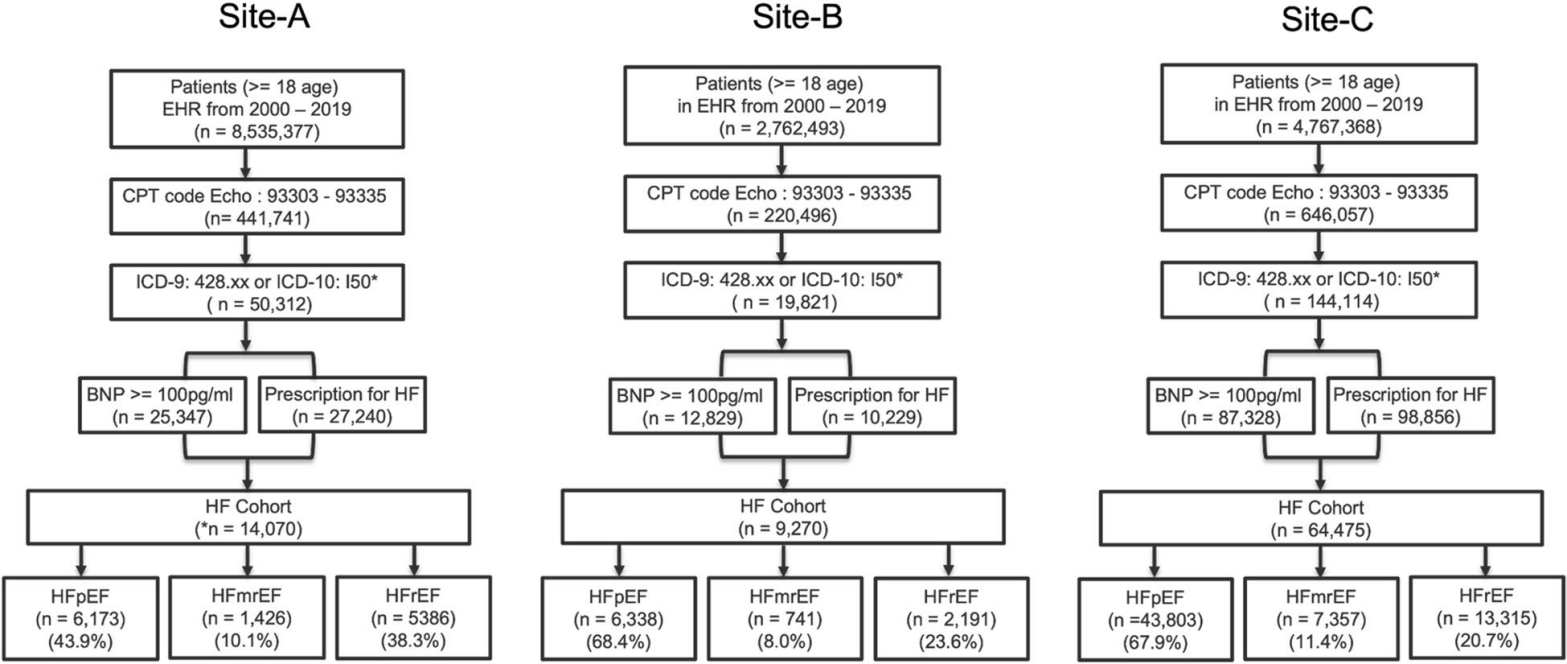
Heart failure subphenotypes at the three sites. For Site-A there were 1085 (7.7%) patients with no EF values available.

Following a previously validated HF algorithm^24^, our inclusion criteria consisted of: (1) All patients 18 years or older from 2000 to 2019 (2) Current Procedural Terminology (CPT) code for echocardiogram (93303 to 93355) (3) International Classification of Disease-Ninth Revision (ICD-9) code diagnosis of HF (any 428.xx) or ICD-10 code diagnosis of HF (any I50*), (4) Either B-type natriuretic peptide (BNP) or pro-BNP (NT-proBNP) values recorded and (5) Prescriptions for HF within 6 months of diagnosis, as illustrated in Fig. 1. Subphenotypes of HF were identified by the EF value assessed within 14 days before the first diagnosis of HF by ICD codes (index date).

**Figure 2:**
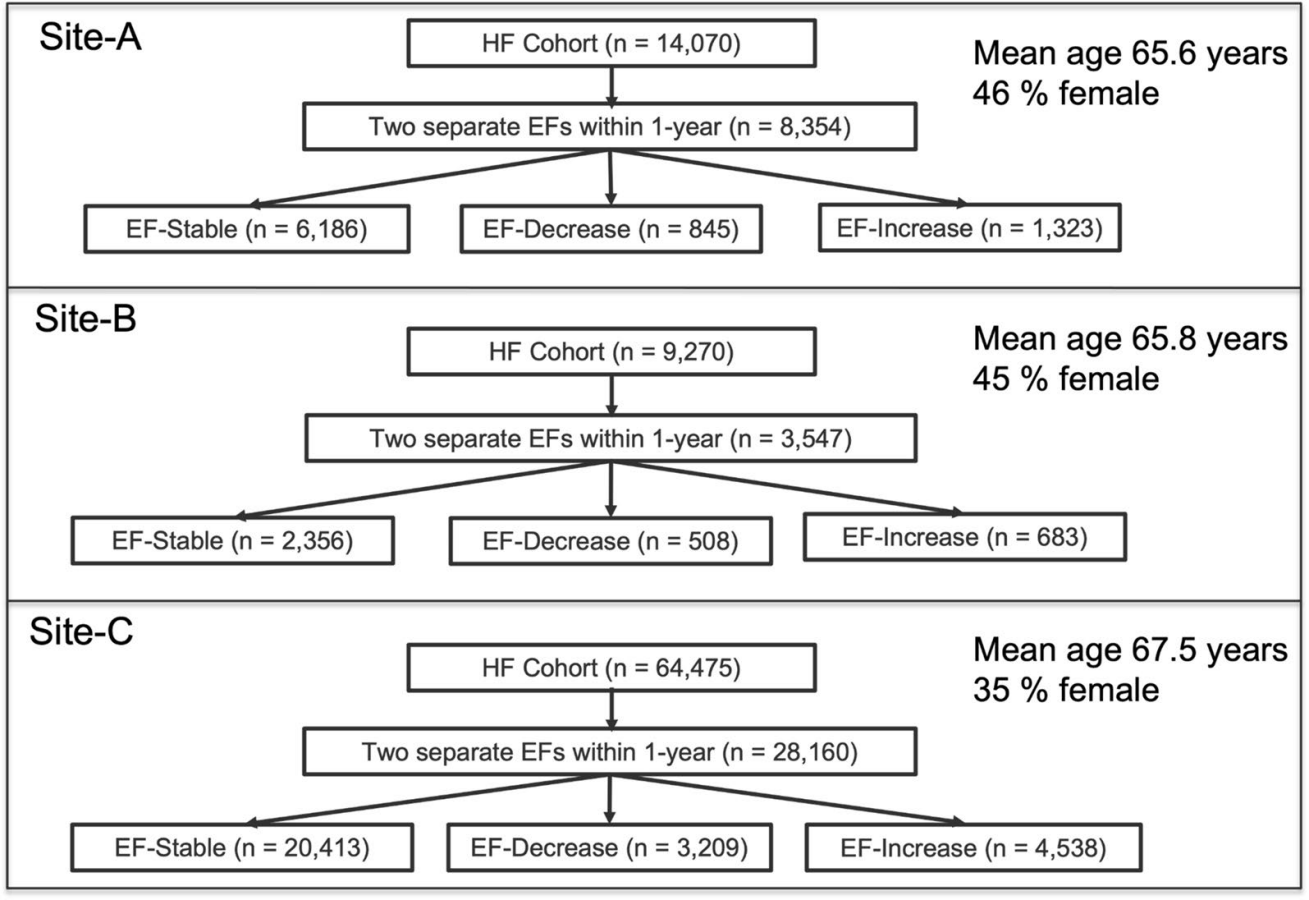
HF patients identified as EF-Stable, EF-Decrease and EF-Increase.

As shown in Fig. 2, from the base HF population defined above, we further identified patients with at least 2 consecutive EF measurements over a 1-year duration. When the same patient had more than 2 EF measurements annually, the first and last assessments were considered in order to calculate the change in EF. The index EF was defined as the first available EF within 14-days before a diagnosis of HF. We took the last EF after 6 months but less than 12 months from the index EF date as the follow-up EF measurement, allowing a minimum of 6 months and maximum 12 months interval between the first and last EF measurements. As defined in Table 1 transitions from HFpEF to HFmrEF and HFpEF to HFrEF, and HFmrEF to HFrEF were pooled and defined as EF-Decrease. Transitions from HFrEF to HFmrEF, HFrEF to HFpEF, and HFmrEF to HFpEF were pooled and defined as EF-Increase. Those patients with no change among EF measurements were pooled and defined as EF-Stable.

**Table 1:** HF patients defined based on categories of decreasing, increasing and stable EF measurements when studying longitudinal changes.

Category	Transitions
EF-Decrease	HFpEF to HFmrEF
	HFpEF to HFrEF
	HFmrEF to HFrEF
EF-Increase	HFrEF to HFmrEF
	HFrEF to HFpEF
	HFmrEF to HFpEF
EF-Stable	HFpEF to HFpEF
	HFrEF to HFrEF
	HFmrEF to HFmrEF

All baseline demographic and clinical data for HF patients including laboratory data, medications, devices, and comorbidities were collected from the corresponding EHRs. Clinicians’ ICD-9 and ICD-10-coded diagnoses were used to define related comorbidities. While querying the data, wildcard searches were used to capture broader categories of each disease (full list available in Supplemental Table S1). Smoking status was classified as ‘former’, ‘current’, ‘never’, or ‘unknown’. Arterial diastolic blood pressure (BP), arterial systolic BP, body mass index (BMI), B-type natriuretic peptide (BNP), diastolic BP, systolic BP, estimated glomerular filtration rate (EGFR), heart rate, hemoglobin, and respiratory rate were the closest measurements recorded before first HF diagnosis. We used RxNorm codes or pharmaceutical classes, and subclasses defined within the EHR (Epic) for identifying medications for ACE inhibitors or ARB, digoxin, platelet inhibitor, nitrate, statin, SGLT2 inhibitor, and string matching in names for diuretic, beta-blocker, and oral anticoagulant. Baseline characteristics of patients according to increasing, decreasing, or stable EF were compared using a Student’s t-test for continuous variables and chi-square test for categorical variables. All statistical analysis was performed using R version 3.6.1^28^.

**Figure 3:**
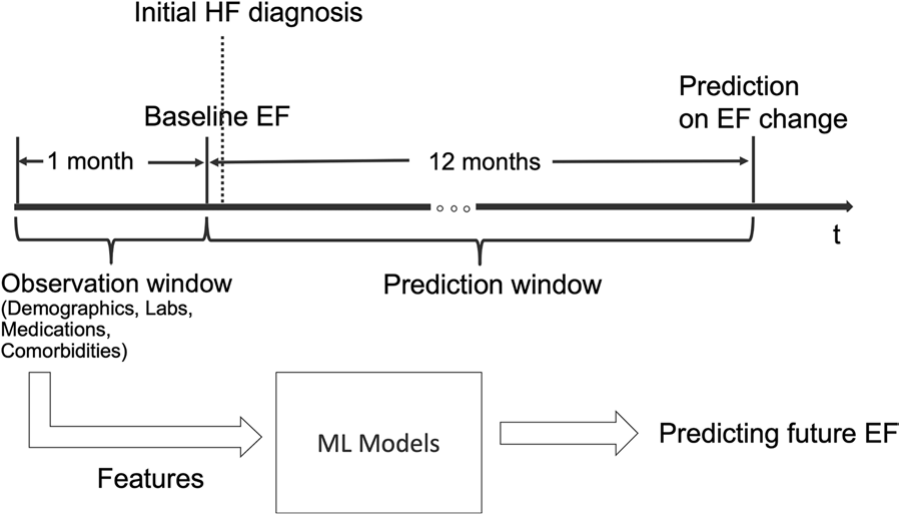
Prediction settings for the ML models.

The baseline characteristics of patients were then used to develop various machine learning models. Even though clinicians generally are interested in looking at characteristics of subgroups of patients whose EF improved (HFrEF to HFmrEF/HFpEF) or declined (HFpEF to HFmrEF/HFrEF and HFmrEF to HFrEF) from baseline values, we used the entire HF cohort for building the models. The expectation was to develop a general purpose model to predict EF changes in HF patients regardless of their initial EF, which could then be trained and tested for any given subset of patients. As shown in Fig. 3, if t is the time of EF measurement before the first HF diagnosis, either during an outpatient or inpatient encounter, we used all the data prior to time t in terms of patient demographics, labs, vitals, comorbidities, and medications for training multiple machine learning models to predict EF change after 1 year. A patient may have multiple EF measurements before and after a HF diagnosis. We used the first EF measurement within 14 days before the HF diagnosis as the baseline EF. The 14-day short period between EF measurement and HF diagnosis was chosen to ensure that the diagnosis was indeed made based on this recent assessment of EF. Some patients may have been missed from inclusion in our analysis by this strict criterion of 14 days. A 1-month look back window was used for lab measurements, vitals, comorbidities, and medications. The ML models included Logistic Regression (LR)^29^, Random Forest (RF)^30^, Support Vector Machines (SVM)^31^, XGBoost^32^, K-Nearest Neighbor (Knn)^33^, and Decision Tree (DT)^34^. All models were trained to predict EF changes in 10, 15, 20, and 30 percentages of increase and decrease between the baseline and follow-up values. An EF increase or decrease of up to 30 percent was chosen based on manual review of select patients. For a HFpEF patient with a baseline EF of 50, a 10 and 30 percent increase in EF would result in a 1-year follow-up EF of 55 and 65, respectively. For a HFrEF patient with a baseline EF of 30, a 10 and 30 percent increase in EF would result in a 1-year follow up EF of 33 and 39, respectively. Similarly, for a HFrEF patient with a baseline EF of 30 a 10 and 30 percent reduction in EF would result in a 1-year follow-up EF of 27 and 21, respectively. Such changes are frequently observed in our patient cohorts.

**Figure 4:**
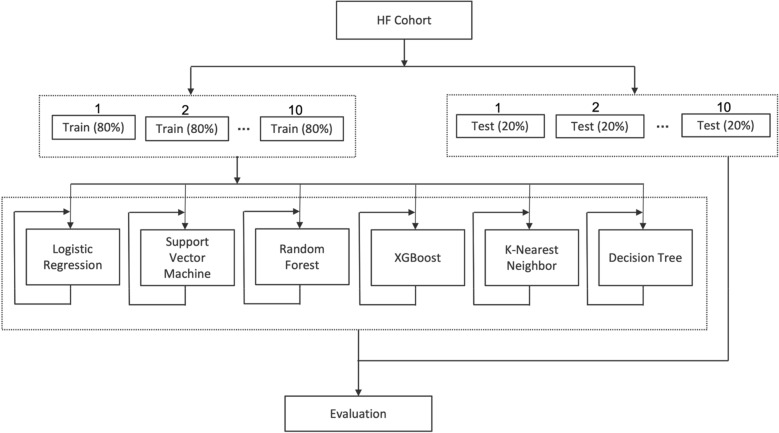
ML models’ setup.

Figure 4 shows the overall strategy we used to develop prediction models. We used a Monte Carlo cross-validation technique to optimize the performance. The HF cohort across all sites was randomly divided into a training (80%) and testing (20%) dataset for each run. An initial investigation of the dataset revealed that the sample is unbalanced between the two classes; patients whose EF values changed by a certain percentage versus patients whose values remained the same. This observation is also supported by the fact that a significantly high percentage of patients remained in their baseline category as compared to patients whose values decreased or increased to other categories as given in Fig. 2. In order to address the imbalance between the classes, the training dataset was further oversampled with randomly selected copies of minority class members. We used the sklearn resample method to bring both classes equal. Ten iterations were performed for each outcome to account for variability among patients in the training and testing datasets. During each run, the training dataset was once again randomly shuffled and trained through 10 cycles, so that during each cycle the model gets to see a different order of training samples. The scikit-learn and XGBoost packages were used to implement various machine learning models. For most cases, we used the pre-built default parameters in scikit-learn and XGBoost. Various parameters from the corresponding packages used in training the models are provided in Supplemental Table S2. For all models we used a classification cutoff of 0.5; a score 0.5 and above is a positive prediction and below is a negative prediction. All models were evaluated in terms of precision, recall and F1-score measures macro averaged over the positive label (i.e., patients whose EF changed in one direction vs. patients whose EF values remained stable or changed in the opposite direction). The sklearn function macro computes f1 for each label and returns the average without considering the proportion for each label in the dataset. An area under the receiver operating characteristic curve (AUC) was also computed to evaluate model confidence. Features from the model that performed the best, were ranked ordered based on their variable importance measure. Data missingness is a challenge for EHR-based analyses. We addressed missing data in the following manner: during the preprocessing step, all continuous variables (such as age, diastolic and systolic blood pressure, BMI, EGFR, heart rate, hemoglobin, and respiratory rate) were first converted into categorical variables. Then, for each categorical variable, we created multiple categories in which a patient’s value could fall. For example, in the case of BMI, we created 5 categories named “BMI_UNDER” for values below 18.5, “BMI_NORMAL” for values between 18.5 and 24.9, “BMI_OVER” for values between 24.9 and 29.9, “BMI_OBESE” for values over 29.9, and “BMI_UNKNOWN” for values missing or NULL. This way patients whose values were missing for a given variable were tagged under the “_unknown” category, and we excluded these “_unknown” categories in building the model. The expectation was to build a model that can work across EHR systems with available data. Each category except the “_unknown” becomes a feature with a value 0 or 1 for the vector representation for a given patient. For age variable the following categories were defined: AGE_BELOW_30, AGE_30_40, AGE_40_50, AGE_50_60, AGE_60_70, AGE_70_80, AGE_80_90, AGE_90_100, and AGE_OVER_100. For continuous clinical variable we categorized them into categorical variables based on standard clinical practices in the United States. For example, in the case of EGFR with a measurement unit of “mL/min/1.73 m2”, we defined six categories EGFR_NORMAL (≥ 90), EGFR_STAGE2 (≥ 60 and < 90), EGFR_STAGE3 (≥ 30 and < 60), EGFR_STAGE4 (≥ 15 and < 30), EGFR_STAGE5 (< 15), and EGFR_UNKNOWN. A total of 62 variables were included in the model development (see Supplemental Table S3).

In order to gain further insights into the prediction performance of EF changes, feature analysis was performed to determine which features are significant contributors to predicting EF changes in patients. We used the built-in feature importance function of XGBoost for this. In XGBoost, the feature relative importance can be measured by several metrics, such as split weight and average gain. We used the feature rankings of weight-based importance after XGBoost fitting.

## Results

As shown in Fig. 1, for Site-A, there were 8,535,377 patients aged 18 years or older in the EHR between the years 2000 and 2019. There were 441,741 patients who had an echocardiogram. There were 50,312 patients with any HF diagnosis. Refining the criteria for elevation of BNP markers within 6 months of HF diagnosis resulted in 25,347 patients. 27,240 patients were prescribed medication from one of the pharma classes of antihypertensive, diuretics, cardiovascular, or one of the pharma subclasses of beta blockers or beta blocker combinations. A union between the above two sets resulted in 14,070 patients that we identified for the HF base cohort, of which only 12,985 patients had EF measurements available. There were 6173 patients with preserved ejection fraction, 1426 patients with mid-range ejection fraction and 5386 patients with reduced ejection fraction at the index date.

For Site-B, there were 2,762,493 patients aged 18 years or older in the EHR between the years 2000 and 2019. There were 220,493 patients who had an echocardiogram. There were 19,821 patients with any HF diagnosis. Criteria for elevation of BNP markers within 6 months of HF diagnosis resulted in 12,829 patients. 10,229 patients were identified as prescribed a medication from one of the drug classes. A union between the above two sets resulted in 9270 patients that we identified for the HF base cohort. There were 6338 patients with preserved ejection fraction, 741 patients with mid-range ejection fraction and 2191 patients with reduced ejection fraction at the index date.

For Site-C, there were 4,767,368 patients aged 18 years or older in the EHR between the years 2000 and 2019. There were 646,057 patients who had an echocardiogram. There were 144,114 patients with any HF diagnosis. Criteria for elevation of BNP markers within 6 months of HF diagnosis resulted in 87,328 patients. 98,856 patients were identified as prescribed a medication from one of the drug classes. A union between the above two sets resulted in 64,475 patients that we identified for the HF base cohort. There were 43,803 patients with preserved ejection fraction, 7357 patients with mid-range ejection fraction and 13,315 patients with reduced ejection fraction at the index date.

We performed further analysis on HF patients whose EF values changed within one year. As shown in Fig. 2, for Site-A, we identified 8354 patients with two or more EF measurements in 1-year. 6186 patients were labeled as EF-Stable, 845 were labeled as EF-Decrease, and 1323 patients were labeled as EF-Increase. Mean age of this cohort was 65.6 years and comprised 46% females. For Site-B, we identified 3547 patients with two or more EF measurements within 1-year. 2356 patients were labeled as EF-Stable, 508 patients were labeled as EF-Decrease, and 683 patients were labeled as EF-increase. Mean age of this cohort was 65.8 years and comprised 45% females. For Site-C, there were 28,160 patients with two separate EF measurements within1-year. 20,413 patients were labeled as EF-Stable, 3209 patients were labeled as EF-Decrease, and 4538 patients were labeled as EF-increase. Mean age of this cohort was 67.5 years and comprised 35% females.

**Figure 5:**
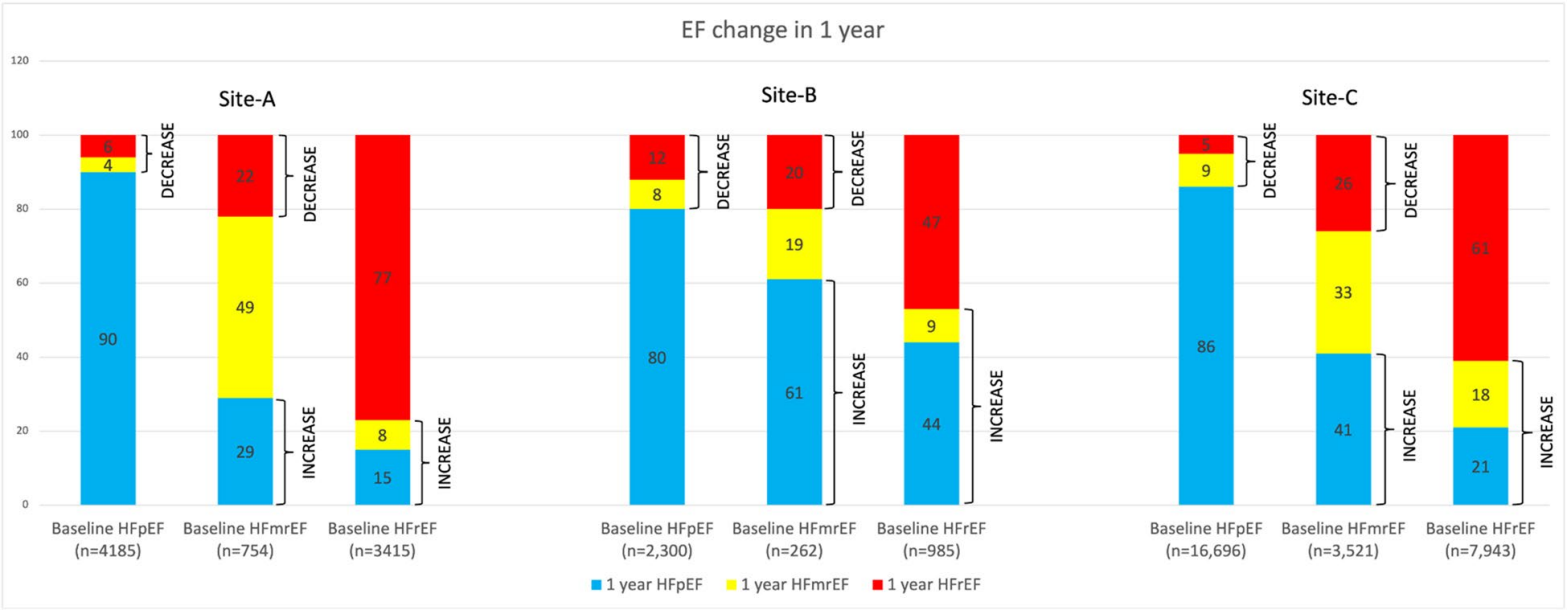
EF changes in 1 year among HF subphenotypes. Each bar segment shows the proportion (%) of patients with changes with stable EFs in each category as references.

Figure 5 shows 1-year longitudinal changes in EF values from the corresponding baseline values for HF patients, across the three sites.

- In Site-A, out of 4185 baseline HFpEF patients, 184 (4%) transitioned to HFmrEF and 246 (6%) transitioned to HFrEF. Of 754 baseline HFmrEF patients, 217 (29%) transitioned to HFpEF and 169 (22%) transitioned to HFrEF. And finally, of 3415 baseline HFrEF patients, 281 (8%) transitioned to HFmrEF and 502 (15%) transitioned to HFpEF.
- In Site-B, out of 2300 baseline HFpEF patients, 178 (8%) patients transitioned to HFmrEF, and 277 (12%) patients transitioned to HFrEF. Of 262 baseline HFmrEF patients, 53 (20%) patients transitioned to HFrEF, and 160 (61%) patients transitioned to HFpEF. And finally, of 985 baseline HFrEF patients, 94(9%) patients transitioned to HFmrEF, and 429 (44%) patients transitioned to HFpEF.
- In Site-C, of 16,696 baseline HFpEF patients, 1497 (9%) patients transitioned to HFmrEF, and 806 (5%) patients transitioned to HFrEF. Of 3521 baseline HFmrEF patients, 906 (26%) patients transitioned to HFrEF, and 1459 (41%) patients transitioned to HFpEF. And finally, of 7943 baseline HFrEF patients, 1452 (18%) patients transitioned to HFmrEF, and 1627 (21%) patients transitioned to HFpEF.

### Baseline characteristics by EF pattern of change

Table 2 and Supplemental Tables S4, S5, and S6 lists patient characteristics according to an increased, decreased, or stable EF for each of the three sites. Since EHR systems vary in their reporting and documentation practices, we were not able to report all variables in a common format. For example, EGFR is documented as a continuous value in Site-B, whereas this is given as categorical values of Stage 1–4. Same was the case with BNP measurements. While Site-B reported this as an absolute value, Site-A and Site-C reported this under “Normal”, “Over” and “Unknown” categories. Also for one of the sites we were able to analyze only a subset of patients (n = 9999) from the initial base HF cohort due to IRB regulations.

**Table 2:** Patient demographic characteristics at the three sites according to an increased, decreased, or stable EF.

Characteristics	EF-Decrease	EF-Increase	EF-Stable
**Site-A**			
n	845	1323	6186
Age (mean (SD))	65.16 (14.83)	60.48 (15.01)	64.26 (15.20)
Sex (%)			
Female	354 (41.9)	497 (37.6)	2844 (46.0)
Male	491 (58.1)	826 (62.4)	3342 (54.0)
Race (%)			
Native American/Alaska Native	2 (0.2)	1 (0.1)	19 (0.3)
Asian	27 (3.2)	44 (3.3)	186 (3.0)
Black/African American	186 (22.0)	293 (22.1)	1511 (24.4)
Hawaiian/Pacific Islander	0 (0.0)	2 (0.2)	6 (0.1)
White	539 (63.8)	844 (63.8)	3772 (61.0)
Declined	33 (3.9)	54 (4.1)	250 (4.0)
Other combination	48 (5.7)	72 (5.4)	363 (5.9)
Unknown	10 (1.2)	13 (1.0)	79 (1.3)
Smoking (%)			
Current	48 (5.7)	87 (6.6)	405 (6.5)
Former	255 (30.2)	380 (28.7)	1674 (27.1)
Never	273 (32.3)	443 (33.5)	2118 (34.2)
Unknown	269 (31.8)	413 (31.2)	1989 (32.2)
BMI (mean (SD))	29.82 (10.52)	30.39 (12.86)	40.94 (455.05)
**Site-B**			
n	507	684	2356
Age (mean (SD))	67.57(15.07)	65.78 (14.84)	69.74(14.51)
Sex (%)			
Female	187 (36.9)	270 (39.5)	1147 (48.7)
Male	320 (63.1)	414 (60.5)	1208 (51.3)
Unknown	0 (0.0)	0 (0.0)	1 (0.0)
Race (%)			
Native American/Alaska Native	3 (0.6)	3 (0.4)	9 (0.4)
Asian	16 (3.2)	11 (1.6)	64 (2.7)
Black/African American	37 (7.3)	78 (11.4)	220 (9.3)
Hawaiian/Pacific Islander	0 (0.0)	0 (0.0)	4 (0.2)
White	125 (24.7)	156 (22.8)	592 (25.1)
Declined	27 (5.3)	46 (6.7)	148 (6.3)
Other combination	94 (18.5)	102 (14.9)	380 (16.1)
Unknown	205 (40.4)	288 (42.1)	939 (39.9)
Smoking (%)			
Current	3 (0.6)	1 (0.1)	20 (0.8)
Former	52 (10.3)	60 (8.8)	280 (11.9)
Unknown	452 (89.2)	623 (91.1)	2,056 (87.3)
BMI (mean (SD))	28.35 (6.24)	28.07 (6.22)	28.83 (7.10)
**Site-C**			
n	3109	3335	3555
Age (mean (SD))	67.42 (13.36)	64.28 (14.51)	66.54 (13.77)
Sex (%)			
Female	1082 (34.8)	1189 (35.7)	1342 (37.7)
Male	2027 (65.2)	2145 (64.3)	2213 (62.3)
Unknown	0 (0.0)	1 (0.0)	0 (0.0)
Race (%)			
Native American/Alaska Native	12 (0.4)	15 (0.4)	19 (0.5)
Asian	28 (0.9)	41 (1.2)	48 (1.4)
Black/African American	113 (3.6)	149 (4.5)	135 (3.8)
Hawaiian/Pacific Islander	5 (0.2)	5 (0.1)	7 (0.2)
White	2875 (92.5)	3027 (90.8)	3243 (91.2)
Declined	16 (0.5)	17 (0.5)	12 (0.3)
Other combination	44 (1.4)	59 (1.8)	65 (1.8)
Unknown	16 (0.5)	22 (0.7)	26 (0.7)
Smoking (%)			
Current	81 (2.6)	134 (4.0)	137 (3.9)
Former	157 (5.0)	214 (6.4)	342 (9.6)
Never	359 (11.5)	359 (10.8)	516 (14.5)
Unknown	2,512 (80.8)	2,628 (78.8)	2,560 (72.0)
BMI (mean (SD))	30.42 (10.52)	32.08 (32.61)	31.86 (12.19)

In Site-A, HF patients with increased EF were younger (mean 60.48, sd:15.01) than those patients whose EF decreased or remained unchanged. HF patients with increased EF also had an average higher heart rate and diastolic blood pressure compared to the other two categories. BNP and hemoglobin measurements were missing for the majority of patients. Patients with decreasing EF had a higher prevalence of stroke and atrial fibrillation (AF) than those with stable or increasing EF.

In Site-B, HF patients with increasing EF were among the youngest. In the overall HF population, the mean age was 68.4 (sd:14.9) years of age, 45% were female, 65% had HFpEF, 7% had HFmrEF, and 28% had HFrEF. Lab measurements such as EGFR and hemoglobin levels were missing in the majority of patients. Also, BNP measurements were missing in 51% of the patient cohort. Among those patients with BNP measurements available, patients with increasing EF were found to have a higher BNP value compared with patients with decreasing or stable EF. Patients with decreasing EF had higher comorbidities (e.g., hypertension, diabetes, ischemic heart disease, peripheral artery disease, anemia) than those with stable or increasing EF. Use of ACE inhibitors, platelet inhibitors, nitrate, statin, and beta blocker were highest among patients whose EF decreased. The use of diuretics was found more in patients with stable EF than patients with decreasing or increasing EF. The use of cardiac resynchronization therapy and implantable cardioverter defibrillator was highest in those with stable EF. The use of beta-blocker is lowest in Site-B. However, given the fact that the p-value (0.1) not statistically significant and the large proportion of patients without beta-blockers, any possible effect of beta-blockers on EF change is unclear.

In Site-C, similar to Site-A and Site-B, HF patients with increased EF were younger (mean 64.28, sd:14.51) than those patients whose EF decreased or remained stable. EF-increase patients had an average higher heart rate and diastolic blood pressure compared to the other two categories. BNP and EGFR measurements were missing for the majority of patients. Use of ACE inhibitors, platelet inhibitors, nitrate, statin, and beta blocker were highest among patients whose EF increased or stable than with patients whose EF decreased. The use of diuretics was found more in EF-increase and EF-stable patients than EF-decrease patients.

### EF change prediction

Table 3 shows the prediction performances of ML models in terms of precision, recall, F1-score, and AUC in classifying patients with 10, 15, 20 and 30 percent increase in EF versus patients with stable or decrease in EF from the baseline values. For all models, performance was generally consistent across all sites. The prediction in terms of precision, recall and F1-score, increases when testing for EF increases from 10 to 30%. The highest performance was observed at 30% EF increase for all models. Generally high precisions and slightly lower recalls were observed resulting in moderate to high F1 scores. The recall observed at Site-A is generally lower than that observed at the other two sites. The AUC values were also found to be improving when predicting EF increases from 10 to 30%, implying all models have higher confidence in predicting instances of a large increase in EF values. Across all sites, XGBoost showed the highest performance in terms of F1-score and AUC. It should be noted that, while other models performed comparably to XGBoost in Site-B and Site-C, the performance of these models was lower in Site-A.

**Table 3:** Performance of various ML models in classifying patients with increased EF values from those patients with either decreased or stable EF over the same period.

Site-A																
	Precision				Recall				F1-Score				AUC			
	10%	15%	20%	30%	10%	15%	20%	30%	10%	15%	20%	30%	10%	15%	20%	30%
LR	84.7	89.1	93.2	96.5	67.3	67.5	68.1	69.6	75.0	76.8	78.7	80.8	0.75	0.77	0.81	0.85
SVM	86.3	89.7	92.8	96.6	63.3	65.1	69.0	69.9	73.0	75.4	79.0	81.1	0.74	0.77	0.80	0.83
RF	84.1	88.7	93.2	97.1	68.7	68.8	68.3	68.0	75.7	77.4	78.8	80.0	0.74	0.77	0.80	0.83
XGBoost	75.9	79.9	84.1	87.8	81.7	83.6	84.9	86.9	78.5	81.5	84.4	87.2	0.72	0.75	0.79	0.83
Knn	76.8	81.9	86.6	90.9	64.0	65.4	67.8	69.9	69.8	72.8	76.0	79.0	0.66	0.69	0.72	0.76
DT	86.3	89.7	92.9	96.6	63.3	65.2	69.0	69.9	73.0	75.4	79.0	81.1	0.73	0.75	0.79	0.82

Site-B																
	Precision				Recall				F1-Score				AUC			
	10%	15%	20%	30%	10%	15%	20%	30%	10%	15%	20%	30%	10%	15%	20%	30%
LR	87.3	91.0	94.6	96.9	82.5	81.1	79.8	82.2	84.9	85.7	86.6	88.9	0.80	0.82	0.85	0.89
SVM	87.4	91.2	94.9	97.0	82.7	80.8	79.3	81.5	85.0	85.6	86.4	88.5	0.79	0.81	0.84	0.89
RF	87.4	91.2	94.8	96.8	82.7	80.8	79.4	83.2	85.0	85.7	86.5	89.6	0.79	0.80	0.84	0.88
XGBoost	83.3	86.4	90.0	92.8	83.5	84.6	85.9	87.5	83.4	85.6	87.9	89.9	0.77	0.80	0.83	0.87
Knn	83.2	87.5	91.3	94.8	73.6	74.7	76.6	78.7	78.0	80.9	83.1	86.0	0.74	0.77	0.80	0.83
DT	87.3	91.2	94.9	97.0	82.8	80.8	79.3	81.1	85.0	85.6	86.4	88.3	0.79	0.82	0.84	0.88

Site-C																
	Precision				Recall				F1-Score				AUC			
	10%	15%	20%	30%	10%	15%	20%	30%	10%	15%	20%	30%	10%	15%	20%	30%
LR	87.2	90.6	92.2	95.5	78.2	79.1	79.8	81.9	82.5	84.5	85.5	88.2	0.87	0.89	0.90	0.91
SVM	91.7	88.2	88.9	94.7	67.3	81.0	86.2	83.6	77.6	83.9	87.5	88.8	0.87	0.89	0.89	0.91
RF	82.7	86.8	89.9	95.0	85.0	85.0	84.3	83.0	83.8	85.9	87.0	88.5	0.86	0.88	0.88	0.90
XGBoost	81.4	84.8	87.0	90.2	84.3	85.0	85.6	87.0	82.9	85.0	86.4	88.6	0.85	0.88	0.89	0.90
Knn	82.3	86.7	88.9	92.6	74.2	74.7	75.0	75.7	78.0	80.2	81.3	83.3	0.81	0.83	0.84	0.85
DT	91.7	88.3	88.9	94.7	67.3	80.9	86.2	83.6	77.6	83.8	87.5	88.8	0.85	0.87	0.88	0.90

Similarly, Table 4 shows prediction performance of various models in classifying patients with a decrease in EF in 10, 15, 20, and 30 percentages from the baseline values versus those patients with either increased or stable EF from their baseline values. Across all sites, except for XGBoost, all other models showed high precision, but low recall resulting in a moderate F1-score. The XGBoost model, on the other hand, showed high precision and recall resulting in a high F1-score across all sites. As in the case of EF increase, prediction performance in terms of recall and precision increases when testing for EF decreases from 10 to 30%. The highest performance observed for 30% EF decrease for all models. However, unlike the EF increase case, the AUC values were generally low with no significant change for the various cases of EF decrease, implying low confidence in models prediction ability. The low AUC is further evidenced from the feature importance analysis shown below. For all EF decrease cases, Site-C showed higher AUC values than the corresponding values in Site-A and Site-B.

**Table 4:** Prediction performance of various ML models in classifying patients with a decrease in EF values from their baseline versus patients whose EF values increased or no changes.

Site-A																
	Precision				Recall				F1-Score				AUC			
	10%	15%	20%	30%	10%	15%	20%	30%	10%	15%	20%	30%	10%	15%	20%	30%
LR	78.9	83.7	88.0	93.0	55.0	56.4	57.7	60.2	64.8	67.3	69.7	73.1	0.56	0.55	0.56	0.57
SVM	79.9	83.8	88.2	93.1	50.5	55.1	59.1	64.8	61.8	66.4	70.8	76.4	0.57	0.56	0.56	0.57
RF	79.9	84.0	88.3	92.9	52.1	53.0	59.0	62.9	63.1	64.8	70.9	74.9	0.57	0.56	0.56	0.57
XGBoost	76.8	82.1	86.8	91.8	89.8	93.6	96.4	98.5	82.7	87.6	91.3	95.0	0.54	0.55	0.55	0.54
Knn	76.7	82.5	87.4	92.0	56.9	59.5	61.5	70.4	65.6	69.3	72.1	79.9	0.50	0.52	0.53	0.52
DT	79.5	83.4	87.7	92.5	53.0	54.8	63.9	76.7	62.6	64.5	73.1	83.4	0.56	0.54	0.54	0.55

Site-B																
	Precision				Recall				F1-Score				AUC			
	10%	15%	20%	30%	10%	15%	20%	30%	10%	15%	20%	30%	10%	15%	20%	30%
LR	77.2	83.0	86.3	91.9	54.2	56.4	58.6	61.4	63.7	67.2	69.8	73.6	0.60	0.62	0.61	0.61
SVM	81.3	84.8	87.0	92.3	41.2	46.3	53.1	61.3	54.6	59.8	65.9	73.7	0.60	0.61	0.61	0.62
RF	81.2	85.0	88.2	92.7	41.9	43.8	49.2	57.4	55.3	57.5	62.7	71.1	0.60	0.61	0.61	0.63
XGBoost	72.2	78.7	83.2	89.9	78.3	83.2	86.1	91.2	74.9	80.8	84.8	90.6	0.56	0.58	0.57	0.56
Knn	73.1	79.7	84.5	90.9	55.7	57.1	59.0	63.8	63.4	65.9	69.1	74.9	0.54	0.55	0.55	0.55
DT	80.5	84.1	86.2	92.2	39.4	43.4	53.7	66.5	52.4	56.5	65.7	77.3	0.59	0.58	0.59	0.61

Site-C																
	Precision				Recall				F1-Score				AUC			
	10%	15%	20%	30%	10%	15%	20%	30%	10%	15%	20%	30%	10%	15%	20%	30%
LR	85.7	88.1	89.8	93.9	61.6	59.5	59.0	57.1	71.7	71.0	71.2	71.0	0.77	0.75	0.74	0.72
SVM	90.0	91.4	91.2	94.3	50.7	48.3	52.9	53.7	64.8	63.2	66.8	68.3	0.77	0.75	0.74	0.72
RF	85.1	87.7	90.1	94.3	60.2	59.6	58.2	55.1	70.2	71.0	70.6	69.5	0.76	0.74	0.73	0.70
XGBoost	74.0	77.7	81.3	87.7	78.4	81.4	84.9	92.7	76.3	79.4	83.0	90.1	0.74	0.73	0.71	0.68
Knn	76.7	80.7	84.2	90.0	64.3	63.3	62.1	65.6	70.1	71.0	71.6	75.9	0.69	0.68	0.66	0.63
DT	90.7	91.4	89.1	93.1	48.6	47.0	57.4	58.2	63.3	61.7	69.1	71.3	0.74	0.72	0.70	0.68

**Figure 6:**
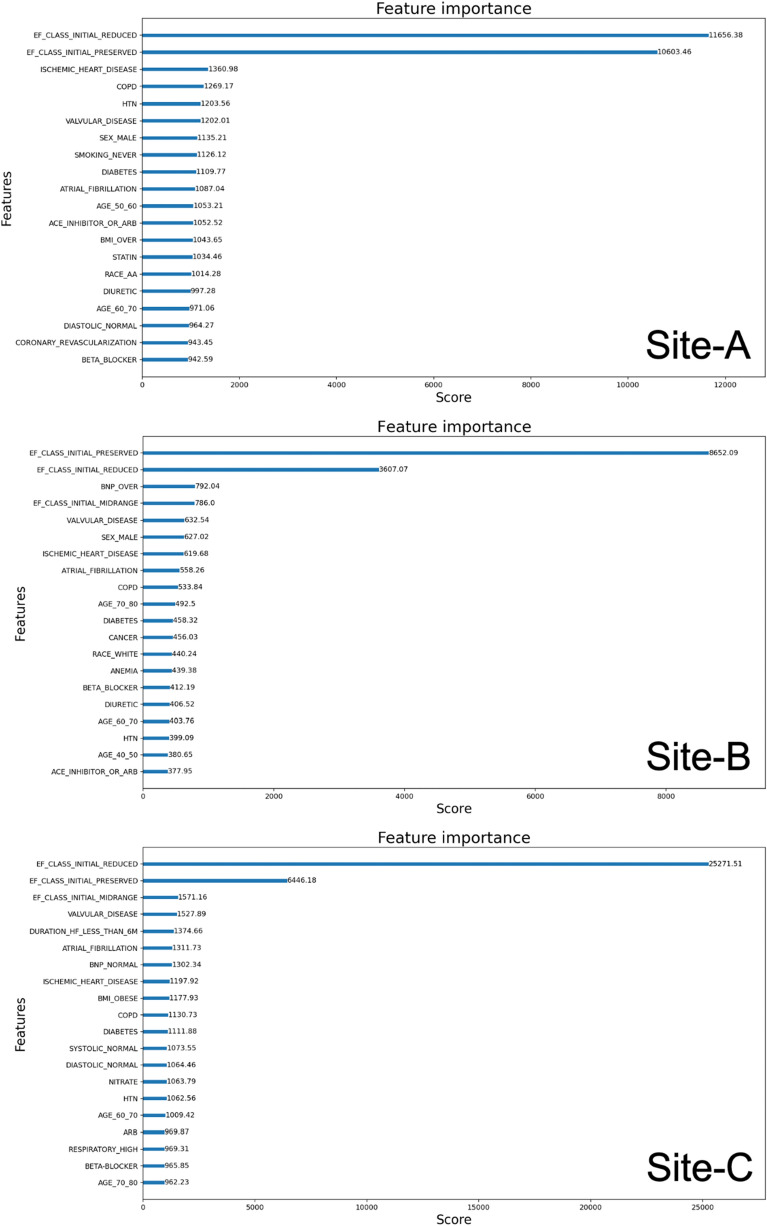
Top features in importance identified for a 30% EF increase at three sites.

We also investigated important features that contribute towards models’ prediction performances. Figure 6 shows the top 20 features and corresponding weights in terms of importance as determined by the XGBoost model for a typical case of 30% EF increase. Similar features were observed in more or less the same order for other cases of EF increase. In general, features such as initial EF, gender, race, heart disease, valvular disease, COPD, BNP, and age between 60 and 70 have significantly higher weights than the remaining feature set. Features that are not listed here have considerably less weight, indicating that they are less relevant and useful in models’ learning improvement. This is also reflected by the high AUC scores measured for these cases at the three sites. Note that in all sites, the top ranked feature initial EF has a significantly higher weight from other features and we found the corresponding AUC score was also higher.

**Figure 7:**
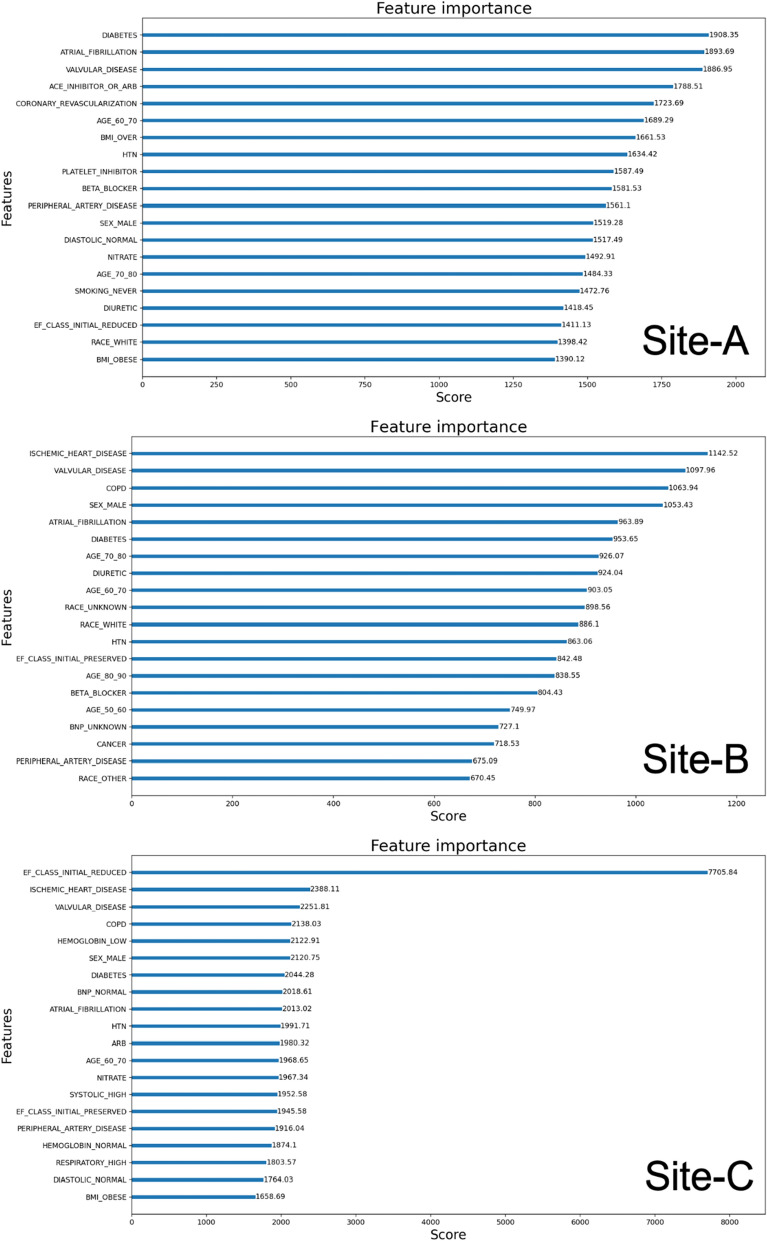
Top 20 features in importance rank identified for EF decrease.

Figure 7 shows the top 20 features and the corresponding weights in terms of importance as determined by the XGBoost model for a typical case of 30% EF decrease. Somewhat similar features were observed in the same or slightly different order for other cases of EF decrease. Unlike the cases of EF increase, the top features have weights that are comparable, and decrease more slowly in their ranks. While diabetes, atrial fibrillation, valvular disease, ACE inhibitors, ischemic heart disease, COPD, sex, and initial EF were found to be important features in all sites, we observed some variations in the order and rank of these features across sites. Also, features not listed here also have some significant weights suggesting their relevance towards contributing to models’ learning. This is also reflected by the low AUC scores measured for these cases across all three sites. Note that in Site-C, the initial EF has a significant weight from other top features and the corresponding AUC score is higher compared to Site-A and Site-B.

## Discussion

Clinical diagnosis of HF requires symptoms and signs as evidenced by laboratory data, radiographic images, and documentation of EF. Thus, development of HF phenotypes in population-based studies is often more challenging than EHR-based cohort identification. EHR data have been used previously to identify HF cohorts. For example, Patel et al. developed and validated an algorithm to identify HFpEF in an EHR system^24^. In this algorithm, the authors used the Veterans Affairs EHR system to identify a cohort of HFpEF using an inclusion criterion of any ICD-9 code of HF (428.xx) and either BNP or aminoterminal pro-BNP (NT-proBNP) values recorded OR diuretic use within one month of diagnosis of HF. EF values were extracted from clinical documents using NLP and used further to identify HFpEF subphenotype. The algorithm was validated through manual chart reviews and had a sensitivity of 88%, specificity of 96%, a positive predictive value of 96%, and a negative predictive value of 87% to identify HFpEF cases. In another study, Tison, et al. developed multiple algorithms for HF cohorts from EHR data, manually reviewed to confirm HF according to Framingham criteria, and evaluated their sensitivity and prediction performances^19^. They reported a sensitivity of 46.3%, specificity of 99.7%, a positive predictive value of 82.5%, and a negative predictive value of 98.4% for an algorithm similar to the one used in the present study. In an extended analysis of a subpopulation of patients with NT-proBNP measured, they reported a sensitivity of 76.3%, specificity of 88.6%, positive predictive value of 82.5% and negative predictive value of 84.1%. The present algorithm differs from the reported one which used an elevated NT-proBNP > 450 pg/ml instead of the BNP > 100 pg/ml in our study. As these above reports clearly demonstrated the validity of the present approach of curating HF cohorts from EHR data, we made no attempt to further validate cohorts through any manual chart review.

In order to compare directly with existing results, we focused our qualitative description of EF changes across various subcategories rather than looking at EF changes in percentage. We found significant differences in the baseline characteristics as well as the longitudinal EF change behavior of the HF cohort in this study with those characteristics reported for the patient cohort in a heart failure registry (SwedeHF) study^14^. The mean age of 65.6 years (Site-A), 65.8 years (Site-B), and 67.5 years (Site-C) in the current study is lower than the mean age of 72 years reported in the SwedeHF study. Also, the current study population has 46% (Site-A), 45% (Site-B) and 35% (Site-C) female patients which is higher than the 31% female patients in the SwedeHF study. In the SwedeHF study of 4942 patients at baseline, 18% had HFpEF, 19% had HFmrEF, and 63% had HFrEF. During follow-up, 21% and 18% of HFpEF patients transitioned to HFmrEF and HFrEF, respectively; 37% and 25% of HFmrEF patients transitioned to HFrEF and HFpEF, respectively; and 16% and 10% of HFrEF patients transitioned to HFmrEF and HFpEF, respectively.

In the present study across all three sites only a small proportion of HFpEF patients observed a decrease in their EF measurements within 1 year. For Site-A this is 10% (4% HFmrEF and 6% HFrEF), for Site-B this is 20% (8% HFmrEF and 12% HFrEF), and for Site-C, this is 14% (9% HFmrEF and 5% HFrEF). In the SwedeHF study, on the other hand, 39% (21% HFmrEF and 18% HFrEF) of HFpEF patients changed to HFmrEF and HFrEF. Across all three sites, a higher proportion of patients initially with mid-range ejection improved their EF measurements as compared to the SwedeHF study. And more significantly, a higher percentage of patients initially with reduced ejection fraction improved their EF values across all three sites compared to the SwedeHF study. For Site-A this is 27% (17% HFpEF and 10% and HFmrEF), for Site-B this is 53% (44% HFpEF and 9% HFmrEF), and for Site-C this is 39% (21% HFpEF and 18% HFmrEF). Whereas in the SwedeHF study, only 26% of HFrEF patients transited to HFpEF and HFmrEF. Median follow-up time in the SwedeHF study was 1.4, which is longer than the 1-year period we studied. Although age and gender were found to be the main contributing factors for the observed differences in EF changes among sites, other factors such as race/ethnicity variations, socio-economics disparity, and differences in treatment options at individual sites may also have contributed to the observed differences.

Accurately predicting EF changes over a period of time in patients with cardiovascular conditions is fundamental to patient-centered care, both in selecting treatment strategies and informing patients as a foundation for shared decision making. Using data from EHR on a cohort of HF patients belonging to the subphenotypes of HFpEF, HFmrEF and HFpEF, this study investigated several machine learning methods to build prediction models for EF assessment. Although most models performed well in predicting EF value changes, the XGBoost model performed the best with good internal validation. Unlike other classifiers attempted in this study, XGBoost is an ensemble method that uses a gradient boosting framework and generally works best in classification problems with missing values. While these models were found to be efficient in predicting both EF increase and decrease, the performance was found to be higher for EF increase. As evident from Fig. 5, across all sites a higher proportion of patients increased their EF values in 1-year compared to patients whose EF decreased. The ML models were able to learn EF increase with a higher confidence than EF decrease and probably helps explain the differences in performance between these two cases. On analyzing the features that are significant in prediction models, we found that initial EF, gender, heart diseases, COPD, valvular diseases, race, and diabetes were factors that had the highest contribution in predicting an increase in EF measurements. Predictors such as gender, ischemic heart disease, HTN, diabetes are in line with previous reports^14,35^. In developing the various ML models our objective was to investigate the viability of a general-purpose model for EF change prediction regardless of patients baseline subphenotype EF category. It is well known that the pathophysiology and responses of patients in subphenotypes are different, and predicting EF change behavior within any given subphenotypes has great clinical significance. We plan to conduct a similar study of EF changes in patients who had baseline EF in only one of the subphenotypes of EF.

Over the years several risk assessment tools were reported for clinical outcomes such as mortality and hospitalization for patients with HF^25,36,37^. Blood urea nitrogen level, BMI, and health status were predictive of death, whereas hemoglobin level, blood urea nitrogen, time since previous HF hospitalization, and health status were predictive of HF hospitalization. The present study, on the other hand, explores specifically EF value changes in patients over a period of time. Previously reported risk assessment tools were developed using data acquired over a relatively small patient population in controlled clinical trials or observational studies, which are often very narrow in their inclusion/exclusion criteria and more generalization of these tools would be problematic. Here we attempted to use longitudinal data on heart failure patients entered in electronic health records which are more readily available to predict EF changes. Patient cohort belongs to a broad spectrum of patients in terms of demographic and clinical attributes. The present study identifies important predictors of EF changes in heart failure patients, which would allow care providers better treatment strategies.

The present study faces some of the same challenges of EHR based cohort analysis as compared to traditional clinical trials or registry based studies^17^. Missing data elements as well as variability of available data elements across systems are issues investigators need to address when using EHR data for clinical research. Traditional clinical trial or registry based cohort studies obtain data designed to address a specific research question, whereas EHR data was collected primarily for clinical care. While the present multi-site study was initiated with the intention of avoiding some of the challenges of a single-site study and to demonstrate the universal adaptability of the present methods and analysis, it still has limitations. In EHR systems, the same information may not be universally available or collected in a standard way. It is widely acknowledged that data capturing and documentation practices vary widely across EHR systems and may depend on factors such as local clinical practices and patient demographics. Even if some of these variables are captured in EHR systems, the data may not have been captured consistently. This is also reflected by the fact that several of our clinical variables are missing at varying proportions at the three participating sites. The ML models trained and tested at the three sites were based on available clinical variables at the corresponding site. Therefore, a model trained on data from one site may not have the same predictive performance when testing on patient data from another site. In other words, the ML methods developed in this study were not externally validated and are portable only in a broader sense but require local training for optimum performance. It would be interesting to see the performance of ML models trained using data from one site and tested on data originated at a different site, which we are planning as an extension of this study. Furthermore, the present study was conducted using data from three large multi-specialty academic medical centers. The adaptability of these methods on other EHR systems with a varying focus on care and clinical practices still needs to be evaluated. Also, if a patient’s EF was measured in an acute phase, the values would be changed by medical treatment or adverse event in that short period. The present study used EF measurements extracted mainly from Echocardiograms by natural language processing and did not capture any information on patients HF treatment phase such as acute or non-acute. Features in ML algorithms can act as significant contributors to a given outcome due to reasons other than biological association. For example, high prevalence or low missingness, etc. of a given feature in the overall sample being analyzed may have an oversized influence in the outcome when compared to features that are low prevalent or missing values and need not necessarily be due to higher biological association. Not all features have the same statistical significance to a given target outcome. Unless we train ML models in a “controlled” feature environment, finding meaningful biological association between features and outcome variables is challenging. The high variability of EHR data across sites makes it very challenging to conduct such training. Although we found certain features were significant contributors in the ML prediction of EF changes, neither the extent to which these features influence a patient's EF measurement is clear, nor do we imply that these features are the ones causing such a change in patients’ EF measurements.

## Conclusion

In this multi-site study, we demonstrated how data from EHRs could be effectively used to develop HF phenotypes and investigate longitudinal changes in EF among HF subphenotypes. We observed significant differences in patients longitudinal EF changes from a previous study using HF registry data. We attribute these differences mainly to the age and gender differences of the study patient population. Data gathered from the longitudinal study were then used to develop various machine learning models to predict EF changes in heart failure patients. Across all three sites, high performances were observed in predicting changes in EF values over a 1-year duration and found ML models were better in predicting EF increases versus EF decreases. As the percentage EF changes increased from 10 to 30%, all model performances were also found to increase. Among various machine learning models, the XGBoost was found to be the best performing model for predicting EF change. The methods developed in this study can be easily portable to other EHR systems, although local training and customization would be needed to ensure optimum performance.

## Supplementary Information

Supplementary Tables.

## Data Availability

The data of this study are not publicly available due to privacy and ethical restrictions. Data to support the findings of this study are available upon reasonable request. Also code for data collection and machine learning models are available on request. Contact the corresponding author for data request.

## Abbreviations

EHR: Electronic health records
HF: Heart failure
EF: Left ventricular ejection fraction
HFpEF: Heart failure with preserved ejection fraction
HFrEF: Heart failure with reduced ejection fraction
HFmrEF: Heart failure with mid-range ejection fraction
