# Supplementary material for: Prediction of left ventricular ejection fraction changes in heart failure patients using machine learning and electronic health records: a multi-site study
Source: Sci Rep. 2023 Jan 6;13:294. doi: 10.1038/s41598-023-27493-8 (PMC9822934; doi:10.1038/s41598-023-27493-8)
Supplement: Supplementary file 1 — Supplementary Tables. [file 41598_2023_27493_MOESM1_ESM.pdf]

Supplemental Material

Table S1. Disease/Condition and corresponding ICD -9/10 codes used in this study

**Disease/Condition**	**ICD9/10 Codes**
hypertension	ICD-10: 'I1[1235]%'; ICD-9: '40[1-5]%'
diabetes	ICD-10: 'E1[0-4]%'; ICD-9: '250%'
ischemic heart disease	ICD-10: 'I2[0-5]%'; ICD-9: '41[0-4]%'
peripheral artery disease	ICD-10: 'I7[0-1]%', 'I73.1%', 'I73.[189]%', 'I77.1%', 'I79.[02]%', 'K55.[189]%', 'Z95.[89]%'; ICD-9: '093.0%', '437.3%', '440%', '441%', '443.[1-9]%', '447.1%', '557.[19]%', 'V43.4%'
anemia	ICD-10: 'D64%','D50.[089]%', 'D5[1-3]%'; ICD-9: '28[0-1]%'
valvular disease	ICD-10: 'A52.0%', 'I0[5-8]%', 'I3[4-9]%', 'I09.[18]%', 'Q23.[0-3]%', 'Z95.[24]%'; ICD-9: '093.2%', '39[4-7]%', '424%', '746.[3-6]%', 'V42.[2-3]%'
chronic obstructive pulmonary disease (COPD)	ICD-10: 'J4[0-7]%', 'I27.[89]%', 'J6[0-7]%', 'J68.4%', 'J70.[13]%'; ICD-9: '416.[89]%', '49[0-9]%', '50[0-5]%', '506.4%', '508.[18]%'
atrial fibrillation	ICD-10: 'I48%'; ICD-9: '427.3%'
stroke	ICD-10: 'G45%', 'I63%', 'J6[0-7]%'; ICD-9: '434%', '435%'
cancer	ICD-10: 'C[0-9][0-9]%', 'D[0-4][0-9]%'; ICD-9: '1[4-9][0-9]%', '2[0-3][0-9]%'

Table S2. Parameters used in training various ML models for predicting EF change

**Disease/Condition**	**ICD9/10 Codes**
Logistic Regression (LR)	penalty='l2',<br>dual=False,<br>tol=0.0001,<br>C=0.1,<br>fit_intercept=True,<br>intercept_scaling=1,<br>class_weight='balanced',<br>random_state=None
Support Vector Machine (SVM)	gamma='auto',<br>probability=True
Random Forest (RF)	n_estimators=100,<br>max_depth=2,<br>random_state=0
XGBoost	learning_rate=0.1,<br>n_estimators=200,<br>max_depth=15,<br>min_child_weight=2,<br>gamma=0,<br>subsample=0.8,<br>colsample_bytree=0.8,<br>objective='binary:logistic',<br>nthread=4,<br>scale_pos_weight=1,<br>seed=27
K-Nearest Neighbors (KNN)	n_neighbors=7,<br>weights='distance',<br>algorithm='auto',<br>leaf_size=30,<br>p=2,<br>metric='minkowski',<br>metric_params=None
Decision Tree (DT)	max_depth=2

Table S3. Features used in developing various ML models for predicting EF change

SEX_MALE, SEX_FEMALE,
RACE_AA, RACE_HAWAIIAN_PI, RACE_WHITE, RACE_NATIVE_ALASKAN, RACE_ASIAN,
AGE_BELOW_30, AGE_30_40, AGE_40_50, AGE_50_60, AGE_60_70, AGE_70_80, AGE_80_90,
AGE_90_100, AGE_OVER_100,
EF_CLASS_INITIAL_PRESERVED, EF_CLASS_INITIAL_MIDRANGE,
EF_CLASS_INITIAL_REDUCED,
BMI_UNDER, BMI_NORMAL, BMI_OVER, BMI_OBESE,
BNP_NORMAL, BNP_OVER,
HEART_RATE_LOW, HEART_RATE_NORMAL, HEART_RATE_HIGH,
HEMOGLOBIN_LOW, HEMOGLOBIN_NORMAL, HEMOGLOBIN_HIGH,
RESPIRATORY_LOW, RESPIRATORY_NORMAL, RESPIRATORY_HIGH,
DIASTOLIC_LOW, DIASTOLIC_NORMAL, DIASTOLIC_HIGH,
SYSTOLIC_LOW, SYSTOLIC_NORMAL, SYSTOLIC_HIGH,
HTN,
DIABETES,
ISCHEMIC_HEART_DISEASE,
PERIPHERAL_ARTERY_DISEASE,
ANEMIA,
VALVULAR_DISEASE,
COPD,
ATRIAL_FIBRILLATION,
STROKE_OR_TIA,
CANCER,
MCRA,
ACE_INHIBITOR_OR_ARB,
DIGOXIN,
PLATELET_INHIBITOR,
NITRATE,
STATIN,
DIURETIC,
BETA_BLOCKER,
ORAL_ANTICOAGULANT,
SGLT2_INHIBITOR,
HF_DEVICE,
CORONARY_REVASCULARIZATION

Table S4. Site-A baseline characteristics of HF cohorts whose EF's were increased, decreased and unchanged during 1-year period

	Category	EF-Decrease	EF-Increase	EF-Stable	p	Missing
n		845	1323	6186		
Age (SD)		65.16 (14.83)	60.48 (15.01)	64.26 (15.20)	<0.001	0
Sex (%)					<0.001	0
	Female	354 (41.9)	497 (37.6)	2844 (46.0)		
	Male	491 (58.1)	826 (62.4)	3342 (54.0)		
Smoking (%)					0.495	0
	Current	48 (5.7)	87 (6.6)	405 (6.5)		
	Former	255 (30.2)	380 (28.7)	1674 (27.1)		
	Never	273 (32.3)	443 (33.5)	2118 (34.2)		
	Unknown	269 (31.8)	413 (31.2)	1989 (32.2)		
Race (%)					0.702	0
	Native	2 (0.2)	1 (0.1)	19 (0.3)		
	Asian	27 (3.2)	44 (3.3)	186 (3.0)		
	Black	186 (22.0)	293 (22.1)	1511 (24.4)		
	Hawaiian	0 (0.0)	2 (0.2)	6 (0.1)		
	White	539 (63.8)	844 (63.8)	3772 (61.0)		
	Declined	33 (3.9)	54 (4.1)	250 (4.0)		
	Other	48 (5.7)	72 (5.4)	363 (5.9)		
	Unknown	10 (1.2)	13 (1.0)	79 (1.3)		
BMI (SD)		29.82 (10.52)	30.39 (12.86)	40.94 (455.05)	0.593	15.1
BNP (%)					<0.001	0
	Normal	20 (2.4)	24 (1.8)	147 (2.4)		
	Over	169 (20.0)	476 (36.0)	1509 (24.4)		
	Unknown	656 (77.6)	823 (62.2)	4530 (73.2)		
Diastolic BP (SD)		71.76 (14.24)	74.57 (15.66)	71.22 (13.86)	<0.001	14.2
EGFR (%)					<0.001	0
	Stage 2	147 (17.4)	364 (27.5)	1243 (20.1)		
	Stage 3	105 (12.4)	201 (15.2)	848 (13.7)		
	Stage 4	24 (2.8)	46 (3.5)	166 (2.7)		
	Stage 5	17 (2.0)	19 (1.4)	67 (1.1)		
	Unknown	552 (65.3)	693 (52.4)	3862 (62.4)		
Heart rate (SD)		80.68 (17.75)	84.55 (19.41)	80.80 (17.24)	<0.001	14.2
Hemoglobin (SD)		11.46 (2.31)	12.35 (2.40)	11.90 (2.38)	<0.001	63.5
Respiratory rate (SD)		18.61 (4.95)	18.77 (3.28)	18.53 (3.56)	0.19	33.7
Systolic BP (SD)		127.63 (23.75)	125.59 (26.66)	126.55 (24.03)	0.21	14.2
Hypertension (%)					0.339	0
	No	213 (25.2)	365 (27.6)	1595 (25.8)		
	Yes	632 (74.8)	958 (72.4)	4591 (74.2)		
Diabetes (%)					0.143	0
	No	558 (66.0)	860 (65.0)	3904 (63.1)		
	Yes	287 (34.0)	463 (35.0)	2282 (36.9)		

Ischemic heart disease (%)					<0.001	0
	No	364 (43.1)	566 (42.8)	2962 (47.9)		
	Yes	481 (56.9)	757 (57.2)	3224 (52.1)		
Peripheral artery disease (%)					0.341	0
	No	616 (72.9)	989 (74.8)	4503 (72.8)		
	Yes	229 (27.1)	334 (25.2)	1683 (27.2)		
Anemia (%)					0.583	0
	No	654 (77.4)	1041 (78.7)	4787 (77.4)		
	Yes	191 (22.6)	282 (21.3)	1399 (22.6)		
Valvular disease (%)					0.6	0
	No	487 (57.6)	734 (55.5)	3501 (56.6)		
	Yes	358 (42.4)	589 (44.5)	2685 (43.4)		
COPD (%)					<0.001	0
	No	571 (67.6)	917 (69.3)	3960 (64.0)		
	Yes	274 (32.4)	406 (30.7)	2226 (36.0)		
Cancer (%)					0.007	0
	No	637 (75.4)	1072 (81.0)	4866 (78.7)		
	Yes	208 (24.6)	251 (19.0)	1320 (21.3)		
MR agonist treatment (%)					0.289	0
	No	845 (100.0)	1321 (99.8)	6183 (100.0)		
	Yes	0 (0.0)	2 (0.2)	3 (0.0)		
ACE inhibitor or ARB (%)					0.211	0
	No	498 (58.9)	775 (58.6)	3498 (56.5)		
	Yes	347 (41.1)	548 (41.4)	2688 (43.5)		
Digoxin (%)					0.042	0
	No	784 (92.8)	1187 (89.7)	5585 (90.3)		
	Yes	61 (7.2)	136 (10.3)	601 (9.7)		
Platelet inhibitor (%)					0.088	0
	No	625 (74.0)	1031 (77.9)	4681 (75.7)		
	Yes	220 (26.0)	292 (22.1)	1505 (24.3)		
Nitrate (%)					0.265	0
	No	707 (83.7)	1075 (81.3)	5128 (82.9)		
	Yes	138 (16.3)	248 (18.7)	1058 (17.1)		
Statin (%)					0.043	0
	No	518 (61.3)	861 (65.1)	3801 (61.4)		
	Yes	327 (38.7)	462 (34.9)	2385 (38.6)		
Diuretic (%)					<0.001	0
	No	633 (74.9)	1016 (76.8)	4389 (71.0)		
	Yes	212 (25.1)	307 (23.2)	1797 (29.0)		
Beta blocker (%)					0.603	0
	No	434 (51.4)	658 (49.7)	3063 (49.5)		
	Yes	411 (48.6)	665 (50.3)	3123 (50.5)		

Oral anticoagulant (%)					0.786	0
	No	690 (81.7)	1094 (82.7)	5071 (82.0)		
	Yes	155 (18.3)	229 (17.3)	1115 (18.0)		
HF Device (%)					0.682	0
	No	803 (95.0)	1264 (95.5)	5919 (95.7)		
	Yes	42 (5.0)	59 (4.5)	267 (4.3)		
Coronary revascularization (%)					0.063	0
	No	210 (24.9)	293 (22.1)	1560 (25.2)		
	Yes	635 (75.1)	1030 (77.9)	4626 (74.8)		
Stroke or TIA (%)					0.013	0
	No	697 (82.5)	1152 (87.1)	5262 (85.1)		
	Yes	148 (17.5)	171 (12.9)	924 (14.9)		
Atrial fibrillation (%)					0.031	0
	No	496 (58.7)	800 (60.5)	3885 (62.8)		
	Yes	349 (41.3)	523 (39.5)	2301 (37.2)		
SGLT2 inhibitor (%)					0.659	0
	No	844 (99.9)	1321 (99.8)	6171 (99.8)		
	Yes	1 (0.1)	2 (0.2)	15 (0.2)		

Table S5. Site-B baseline characteristics of HF cohorts whose EF's were increased, decreased and unchanged during 1-year period.

	Category	EF-Decrease	EF-Increase	EF-Stable	p	Missing
n		507	684	2356		
Age (SD)		67.57 (15.07)	65.78 (14.84)	69.74 (14.51)	<0.001	0
Sex (%)					<0.001	0
	Female	187 (36.9)	270 (39.5)	1147 (48.7)		
	Male	320 (63.1)	414 (60.5)	1208 (51.3)		
	Unknown	0 (0.0)	0 (0.0)	1 (0.0)		
HF Type					<0.001	0
	HFpEF	453(89.3)	0(0)	1844(78.3)		
	HFmrEF	54(10.7)	158(23.1)	49(2.1)		
	HFrEF	0(0)	526(76.9)	463(19.6)		
Duration of heart failure (%)					0.031	0
	<6 M	377 (74.4)	551 (80.6)	1808 (76.7)		
	>=6 M	130 (25.6)	133 (19.4)	548 (23.3)		
Smoking status (%)					0.043	0
	Current	3 (0.6)	1 (0.1)	20 (0.8)		
	Former	52 (10.3)	60 (8.8)	280 (11.9)		
	Unknown	452 (89.2)	623 (91.1)	2056 (87.3)		
Race (%)					0.297	0
	Native	3 (0.6)	3 (0.4)	9 (0.4)		
	Asian	16 (3.2)	11 (1.6)	64 (2.7)		
	Black	37 (7.3)	78 (11.4)	220 (9.3)		
	Hawaiian	0 (0.0)	0 (0.0)	4 (0.2)		
	White	125 (24.7)	156 (22.8)	592 (25.1)		
	Declined	27 (5.3)	46 (6.7)	148 (6.3)		
	Other	94 (18.5)	102 (14.9)	380 (16.1)		
	Unknown	205 (40.4)	288 (42.1)	939 (39.9)		
Arterial diastolic BP (SD)		91.00 (NA)	105.00 (NA)	70.33 (18.82)	NA	99.9
Arterial systolic BP (SD)		134.00 (NA)	128.00 (NA)	151.33 (30.83)	NA	99.9
BMI (SD)		28.35 (6.24)	28.07 (6.22)	28.83 (7.10)	0.595	85.7
BNP (pg/mL)(SD)		1336.50 (1702.02)	1934.79 (3210.28)	3460.45 (72999.43)	0.862	55.9
Diastolic BP (SD)		71.31 (12.73)	72.38 (13.17)	72.45 (11.47)	0.581	70.5
EGFR (mL/min/1.73 m2) (SD)		42.00 (19.08)	46.43 (19.65)	46.05 (17.57)	0.543	93
Heart Rate (SD)		95.75 (29.58)	89.00 (6.48)	91.50 (21.73)	0.904	99.3

Hemoglobin (g/dL)(SD)		33.55 (1.98)	33.37 (1.70)	33.51 (1.34)	0.095	15.4
Respiratory rate (SD)		17.20 (9.70)	16.89 (8.75)	17.11 (6.16)	0.939	80.5
Systolic BP (SD)		126.54 (22.66)	128.22 (24.15)	129.32 (21.65)	0.369	70.1
Hypertension (%)					0.028	0
	No	400 (78.9)	571 (83.5)	1859 (78.9)		
	Yes	107 (21.1)	113 (16.5)	497 (21.1)		
Diabetes (%)					0.003	0
	No	355 (70.0)	520 (76.0)	1635 (69.4)		
	Yes	152 (30.0)	164 (24.0)	721 (30.6)		
Ischemic heart disease (%)					0.001	0
	No	186 (36.7)	296 (43.3)	1082 (45.9)		
	Yes	321 (63.3)	388 (56.7)	1274 (54.1)		
Peripheral artery disease (%)					0.162	0
	No	411 (81.1)	576 (84.2)	1910 (81.1)		
	Yes	96 (18.9)	108 (15.8)	446 (18.9)		
Anemia (%)					0.082	0
	No	390 (76.9)	549 (80.3)	1795 (76.2)		
	Yes	117 (23.1)	135 (19.7)	561 (23.8)		
Valvular disease (%)					0.031	0
	No	318 (62.7)	401 (58.6)	1330 (56.5)		
	Yes	189 (37.3)	283 (41.4)	1026 (43.5)		
COPD (%)					0.001	0
	No	343 (67.7)	468 (68.4)	1452 (61.6)		
	Yes	164 (32.3)	216 (31.6)	904 (38.4)		
Cancer (%)					<0.001	0
	No	412 (81.3)	561 (82.0)	1773 (75.3)		
	Yes	95 (18.7)	123 (18.0)	583 (24.7)		
MR agonist treatment (%)					0.706	0
	No	505 (99.6)	683 (99.9)	2349 (99.7)		
	Yes	2 (0.4)	1 (0.1)	7 (0.3)		
ACE inhibitor or ARB (%)					0.324	0
	No	317 (62.5)	453 (66.2)	1494 (63.4)		
	Yes	190 (37.5)	231 (33.8)	862 (36.6)		
Digoxin (%)					0.935	0
	No	452 (89.2)	614 (89.8)	2112 (89.6)		
	Yes	55 (10.8)	70 (10.2)	244 (10.4)		
Platelet inhibitor (%)					0.002	0
	No	428 (84.4)	623 (91.1)	2078 (88.2)		
	Yes	79 (15.6)	61 (8.9)	278 (11.8)		

Nitrate (%)					0.108	0
	No	440 (86.8)	620 (90.6)	2088 (88.6)		
	Yes	67 (13.2)	64 (9.4)	268 (11.4)		
Statin (%)					0.077	0
	No	323 (63.7)	478 (69.9)	1571 (66.7)		
	Yes	184 (36.3)	206 (30.1)	785 (33.3)		
Diuretic (%)					0.034	0
	No	326 (64.3)	463 (67.7)	1468 (62.3)		
	Yes	181 (35.7)	221 (32.3)	888 (37.7)		
Beta blocker (%)					0.1	0
	No	293 (57.8)	430 (62.9)	1380 (58.6)		
	Yes	214 (42.2)	254 (37.1)	976 (41.4)		
Oral anticoagulant (%)					0.052	0
	No	442 (87.2)	617 (90.2)	2043 (86.7)		
	Yes	65 (12.8)	67 (9.8)	313 (13.3)		
HF device (%)					0.139	0
	No	456 (89.9)	622 (90.9)	2176 (92.4)		
	Yes	51 (10.1)	62 (9.1)	180 (7.6)		
Coronary revascularization (%)					0.336	0
	No	494 (97.4)	671 (98.1)	2287 (97.1)		
	Yes	13 (2.6)	13 (1.9)	69 (2.9)		
Stroke or TIA (%)					0.013	0
	No	465 (91.7)	650 (95.0)	2160 (91.7)		
	Yes	42 (8.3)	34 (5.0)	196 (8.3)		
Atrial fibrillation (%)					0.111	0
	No	352 (69.4)	459 (67.1)	1528 (64.9)		
	Yes	155 (30.6)	225 (32.9)	828 (35.1)		
SGLT2 inhibitor (%)					0.603	0
	No	507 (100.0)	684 (100.0)	2354 (99.9)		
	Yes	0 (0.0)	0 (0.0)	2 (0.1)		

Table S6. Site-C baseline characteristics of HF cohorts whose EF’s were increased, decreased and unchanged during 1-year period.

	Category	EF-Decrease	EF-Increase	EF-Stable	p	Missing
n		3109	3335	3555		
Age (SD)		67.42 (13.36)	64.28 (14.51)	66.54 (13.77)	<0.001	0
Sex (%)					0.068	0
	Female	1082 (34.8)	1189 (35.7)	1342 (37.7)		
	Male	2027 (65.2)	2145 (64.3)	2213 (62.3)		
	Unknown	0 (0.0)	1 (0.0)	0 (0.0)		
Smoking (%)					<0.001	0
	Current	81 (2.6)	134 (4.0)	137 (3.9)		
	Former	157 (5.0)	214 (6.4)	342 (9.6)		
	Never	359 (11.5)	359 (10.8)	516 (14.5)		
	Unknown	2512 (80.8)	2628 (78.8)	2560 (72.0)		
Race (%)					0.561	0
	Native	12 (0.4)	15 (0.4)	19 (0.5)		
	Asian	28 (0.9)	41 (1.2)	48 (1.4)		
	Black	113 (3.6)	149 (4.5)	135 (3.8)		
	Hawaiian	5 (0.2)	5 (0.1)	7 (0.2)		
	White	2875 (92.5)	3027 (90.8)	3243 (91.2)		
	Declined	16 (0.5)	17 (0.5)	12 (0.3)		
	Other	44 (1.4)	59 (1.8)	65 (1.8)		
	Unknown	16 (0.5)	22 (0.7)	26 (0.7)		
BMI (SD)		30.42 (10.52)	32.08 (32.61)	31.86 (12.19)	0.024	31.3
BNP (%)					<0.001	0
	Normal	34 (1.1)	24 (0.7)	58 (1.6)		
	Over	889 (28.6)	1364 (40.9)	1513 (42.6)		
	Unknown	2186 (70.3)	1947 (58.4)	1984 (55.8)		
Diastolic BP (SD)		70.78 (12.77)	74.47 (14.47)	71.15 (20.35)	<0.001	5.1
EGFR (%)					0.014	0
	Normal	2 (0.1)	2 (0.1)	3 (0.1)		
	Stage 2	23 (0.7)	39 (1.2)	50 (1.4)		
	Stage 3	44 (1.4)	40 (1.2)	79 (2.2)		
	Stage 4	5 (0.2)	9 (0.3)	7 (0.2)		
	Stage 5	1 (0.0)	4 (0.1)	4 (0.1)		
	Unknown	3034 (97.6)	3241 (97.2)	3412 (96.0)		
Heart rate (SD)		74.59 (16.51)	83.55 (24.22)	76.61 (25.10)	<0.001	2.7
Hemoglobin (g/dL) (SD)		12.77 (2.11)	13.31 (2.08)	12.81 (2.12)	<0.001	17.9
Respiratory rate (SD)		28.51 (24.44)	28.91 (13.96)	30.00 (34.86)	0.191	42.6
Systolic BP (SD)		127.31 (22.05)	123.11 (22.53)	124.03 (22.34)	<0.001	5
Hypertension (%)					<0.001	0
	No	1570 (50.5)	1491 (44.7)	1362 (38.3)		
	Yes	1539 (49.5)	1844 (55.3)	2193 (61.7)		
Diabetes (%)					<0.001	0

	No	2346 (75.5)	2515 (75.4)	2326 (65.4)		
	Yes	763 (24.5)	820 (24.6)	1229 (34.6)		
Ischemic heart disease (%)					<0.001	0
	No	1679 (54.0)	1641 (49.2)	1536 (43.2)		
	Yes	1430 (46.0)	1694 (50.8)	2019 (56.8)		
Peripheral artery disease (%)					<0.001	0
	No	2408 (77.5)	2662 (79.8)	2475 (69.6)		
	Yes	701 (22.5)	673 (20.2)	1080 (30.4)		
Anemia (%)					<0.001	0
	No	2748 (88.4)	2929 (87.8)	2982 (83.9)		
	Yes	361 (11.6)	406 (12.2)	573 (16.1)		
Valvular disease (%)					<0.001	0
	No	1433 (46.1)	1494 (44.8)	1384 (38.9)		
	Yes	1676 (53.9)	1841 (55.2)	2171 (61.1)		
COPD (%)					<0.001	0
	No	2375 (76.4)	2379 (71.3)	2298 (64.6)		
	Yes	734 (23.6)	956 (28.7)	1257 (35.4)		
Cancer (%)					<0.001	0
	No	2326 (74.8)	2679 (80.3)	2712 (76.3)		
	Yes	783 (25.2)	656 (19.7)	843 (23.7)		
MR agonist treatment (%)					<0.001	0
	No	2949 (94.9)	3017 (90.5)	3106 (87.4)		
	Yes	160 (5.1)	318 (9.5)	449 (12.6)		
Ace inhibitor or ARB (%)					<0.001	0
	No	2319 (74.6)	2063 (61.9)	2157 (60.7)		
	Yes	790 (25.4)	1272 (38.1)	1398 (39.3)		
Digoxin (%)					<0.001	0
	No	2926 (94.1)	2962 (88.8)	3136 (88.2)		
	Yes	183 (5.9)	373 (11.2)	419 (11.8)		
Platelet inhibitor (%)					<0.001	0
	No	2813 (90.5)	2882 (86.4)	2928 (82.4)		
	Yes	296 (9.5)	453 (13.6)	627 (17.6)		
Nitrate (%)					<0.001	0
	No	2046 (65.8)	1810 (54.3)	1781 (50.1)		
	Yes	1063 (34.2)	1525 (45.7)	1774 (49.9)		
Statin (%)					<0.001	0
	No	2218 (71.3)	2089 (62.6)	1884 (53.0)		
	Yes	891 (28.7)	1246 (37.4)	1671 (47.0)		
Diuretic (%)					<0.001	0
	No	2320 (74.6)	1777 (53.3)	1822 (51.3)		
	Yes	789 (25.4)	1558 (46.7)	1733 (48.7)		
Beta blocker (%)					<0.001	0
	No	2189 (70.4)	1672 (50.1)	1792 (50.4)		

	Yes	920 (29.6)	1663 (49.9)	1763 (49.6)		
Oral anticoagulant (%)					<0.001	0
	No	2911 (93.6)	2944 (88.3)	2948 (82.9)		
	Yes	198 (6.4)	391 (11.7)	607 (17.1)		
HF device (ICD or CRT) (%)					0.015	0
	No	3019 (97.1)	3256 (97.6)	3429 (96.5)		
	Yes	90 (2.9)	79 (2.4)	126 (3.5)		
Coronary revascularization (%)					0.095	0
	No	2825 (90.9)	3034 (91.0)	3185 (89.6)		
	Yes	284 (9.1)	301 (9.0)	370 (10.4)		
Stroke or TIA (%)					0.004	0
	No	2909 (93.6)	3152 (94.5)	3290 (92.5)		
	Yes	200 (6.4)	183 (5.5)	265 (7.5)		
Atrial fibrillation (%)					<0.001	0
	No	2198 (70.7)	2018 (60.5)	2227 (62.6)		
	Yes	911 (29.3)	1317 (39.5)	1328 (37.4)		
SGLT2 inhibitor (%)					0.603	0
	No	3107 (99.9)	3331 (99.9)	3553 (99.9)		
	Yes	2 (0.1)	4 (0.1)	2 (0.1)		
